# Supplementary material for: Multidrug-Resistant Bacteria in a COVID-19 Hospital in Zagreb
Source: Pathogens. 2023 Jan 10;12(1):117. doi: 10.3390/pathogens12010117 (PMC9863123; doi:10.3390/pathogens12010117)
Supplement: Supplementary file 1 [file pathogens-12-00117-s001.zip › pathogens-2115013-supplementary.pdf]

Supplementary material

LABORATORY ANALYSIS OF NON-INVASIVE ISOLATES

ENTEROBACTERALES

All seven isolates (five *K. pneumoniae* and two ***E. coli***) were uniformly resistant to amoxicillin, expanded-spectrum cephalosporins or ESC (ceftazidime, cefotaxime, ceftriaxone) and all except one (86%) to amoxicillin/clavulanate, cefepime, gentamicin and ciprofloxacin. Moderate resistant rates of 57% (n=4) were observed for piperacillin/tazobactam, imipenem, meropenem and ertapenem (all *K. pneumoniae*). Resistance to colistin was observed in three *K. pneumoniae* isolates (43%). All isolates exhibited positive double-disk synergy test and inhibitor based test with clavulanic acid indicating ESBL production. CIM and Hodge tests were positive in four carbapenem resistant *K. pneumoniae* isolates being suspicious for carbapenemase production.

Four *K. pneumoniae* isolates transferred ertapenem resistance to *E. coli* recipient strain with the frequency ranging from $1.7\times10^{-5}$ to $7.6\times10^{-3}$. Resistance to non-β-lactam antibiotics was not cotransferred alongside with ertapenem resistance. Four *K. pneumoniae* isolates tested positive for $bla_{\text{OXA-48}}$ and $bla_{\text{CTX-M-15}}$ genes while two *E. coli* and one *K. pneumonie* yielded product only with primers specific for $bla_{\text{CTX-M-15}}$ genes. $bla_{\text{OXA-48}}$ genes were found also in the respective transconjugant strains. IS*1999* preceded $bla_{\text{OXA-48}}$ genes.

$bla_{\text{OXA-48}}$ genes were carried by IncL plasmid whereas IncFIIs was found as additional plasmid in *K. pneumoniae* isolates, but was not found in the transconjugant plasmids.

Results are shown in Supplementary Table S1.

All patients with invasive infections due to *A. baumannii* had also the same strain with identical resistance patterns in surveillance cultures (nasopharyngeal swab, throat swab, axill swab, stool, rectum swab). The non-invasive isolates were not further analysed.

GRAM-POSITIVE BACTERIA

The urinary MRSA isolate exhibited resistance to penicillin, oxacillin, cotrimoxazole and ciprofloxacin but remained susceptible to gentamicin, vancomycin, teicoplanin and linezolid as shown in Supplementary Table S2.

The both VRE isolates exhibited susceptibility to linezolid and daptomycin only (Supplementary Table S3).

| **Strain** | **specimen** | **Category** | **ESBL** | **Hodge/ CIM** | **AMX** | **AMC** | **TZP** | **CAZ** | **CTX** | **CRO** | **FEP** | **IPM** | **MEM** | **ERT** | **GM** | **CIP** | **COL** | **BL** |
|---|---|---|---|---|---|---|---|---|---|---|---|---|---|---|---|---|---|---|
| *K. pneumoniae* 5379 | Wound swab | XDR | + | ++ | >128 | >128 | >128 | >128 | >128 | >128 | 32 | 128 | >128 | R | 32 | 16 | 16 | OXA-48 CTX-M-15 |
| *K. pneumoniae* 308504 | urine | MDR | + | ++ | >128 | >128 | >128 | >128 | >128 | >128 | 64 | 8 | 16 | R | 16 | 64 | 1 | OXA-48 CTX-M-15 |
| *K. pneumoniae* 20525 | Rectum swab | XDR | + | ++ | >128 | >128 | >128 | >128 | >128 | >128 | 32 | 128 | 128 | R | 64 | 128 | 32 | OXA-48 CTX-M-15 |
| *K. pneumoniae* 4836 | urine | XDR | + | ++ | >128 | >128 | >128 | 64 | >128 | >128 | 16 | 16 | 16 | R | 32 | 32 | 4 | OXA-48 CTX-M-15 |
| *K. pneumoniae* | urine | MDR | + | -- | >128 | >128 | >128 | 64 | >128 | >128 | 16 | 0,25 | 0,12 | S | 16 | 32 | 0,5 | CTX-M-15 |
| *E. coli 1993* | Throat swab | MDR | + | -- | >128 | 32 | 8 | 32 | >128 | >128 | 16 | 0.5 | 0.12 | S | 16 | 2 | 0.25 | CTX-M-15 |
| *E. coli* 12895 | urine | MDR | + | -. | >128 | 16 | 16 | >128 | >128 | >128 | 32 | 2 | 0.25 | S | 2 | 32 | 0.12 | CTX-M-15 |

**Supplementary Table S1.** Minimum inhibitory concentrations, susceptibiltiy category, phenotypic tests for beta-lactamase detection and β-lactamase content of Enterobacterales isolates.

Abbreviations: AMX-amoxycillin; AMC-amoxycillin/clavulanic acid; TZP-piperacillin/tazobactam; CAZ-ceftazidime; CTX-cefotaxime; CRO- ceftriaxone; FEP-cefepime; IMI-imipenem; MEM-meropenem; ERT-ertapenem; GM-gentamicin; CIP-ciprofloxacin; COL-colistin; ESBL-inhibitor based test with clavulanic acid for detection of extended-spectrum beta-lactamases;Amp-C-inhibitor based test with phenyloboronic acid for detection of AmpC beta-lactamases; BL-beta-lactamase content; CIM-carbapenem inactivation method

| | PROTOCOL NUMBER | Date | SPECIMEN | PEN | OX | SXT | RIF | GM | CIP | VAN | TEIC | LZD |
|---|---|---|---|---|---|---|---|---|---|---|---|---|
| 4 | 318442 | 31.12. 2020. | URINE | R | R | S | S | S | R | S (0.5) | S (0.5) | S |

**Suppementary Table S2.** Antibiotic susceptibility of methicillin-resistant *Staphylococcus aures* (MRSA)

Abbreviations: Pen-penicillin; OX-oxacillin; SXT: sulphamethoxazole/trimethoprim; RIF-rifapicin; GM- gentamicin; CIP-ciprofloxacin; VAN-vancomycin; TEIC-teicoplanin; LZD-linezolid; for vancomycin and teicoplanin MIC value is shown; ETA-endotracheal aspirate; BC-blood culture

| | Protocol number | SPECIMEN | AMP | VAN | TEIC | LZD |
|---|---|---|---|---|---|---|
| 1 | 174596 | STOOL | R | R (128) | R (128) | S |
| 2 | 5379 | WOUND SWAB | R | R (128) | R (128) | S |

**Supplementary Table S3.** Antibiotic susceptibilities of vancomycin resistant *Enterococcus faecalis* (VRE) isolates

Abbreviations: AMP-ampicillin; VAN-vancomycin; TEIC-teicoplanin; for vancomycin and teicoplanin MIC value is shown; LZD-linezolid
